# Effect of Hypodermic Needle Versus Safety Lancet on the Fear and Anxiety of Needle Prick Among Undergraduate Medical Students During Hematology Practical: A Cohort Study From a Resource-Limited Setting

**DOI:** 10.7759/cureus.27458

**Published:** 2022-07-29

**Authors:** Jyotirmoy Biswas, Arkadeep Dhali, Sumana Panja, Kankana Karpha, Siddhartha Nath, Gopal Krishna Dhali

**Affiliations:** 1 Department of Physiology, College of Medicine and Sagor Dutta Hospital, Kolkata, IND; 2 Department of Gastrointestinal Surgery, School of Digestive and Liver Diseases, Institute of Postgraduate Medical Education and Research, Kolkata, IND; 3 Department of Physiology, College of Medicine and Sagore Dutta Hospital, Kolkata, IND; 4 Department of Gastroenterology, School of Digestive and Liver Diseases, Institute of Postgraduate Medical Education and Research, Kolkata, IND

**Keywords:** belonephobia, lancet, anxiety, fear, hematology practical, needle prick

## Abstract

Background

Most of the government-sponsored medical teaching institutions in India do not provide safety lancets, and hence, the students are compelled to use hypodermic needles. These needles are widely unpopular among students due to the potential hazards (pain, fear, anxiety) associated with them. This study aims to compare any difference in fear and anxiety associated with finger pricking with a hypodermic needle and a safety lancet.

Methods

This is a prospective cohort study. The current study included data from first-year undergraduate medical students for academic sessions 2021-22.

Results

A total of 121 students participated in the study. Eighty-two (67.8%) participants were male. 41/82 male participants and 20/39 female participants were allocated to the intervention cohort. 111 (91.7%) pricked their fingers by themselves. The sight of others pricking during the experiment (17 versus 5, p=0.004) and the thought of pain while pricking (36 versus 16, p<0.001) was significantly associated more with the use of a hypodermic needle compared to a safety lancet. There was no significant difference in students perceiving the smell of the hematology laboratory (4 versus 1, p=0.165) and the sight of blood (9 versus 3, p=0.064) as a factor influencing their fear and anxiety in both cohorts. There was no gender difference in the perception of these factors.

Symptoms were significantly lower in the intervention cohort compared to the control cohort (8 versus 20, odds ratio 0.302, p=0.008). The most common symptom experienced was excessive sweating (n=22, 18.18%), followed by drying of the mouth (n=12, 9.91%). There was a significant difference in pain scores between the intervention and the control cohorts. There was no significant difference in pain scores among male and female subjects.

Conclusion

A considerable difference between the two cohorts was found. The use of lancets can be proposed to medical teaching institutions for psychological advantage, pain reduction, and overall better quality of the process.

## Introduction

Hands-on experience in hematology practical has been an integral part of physiology education in undergraduate medical training [1]. Not only does it familiarise students with technical and experimental aspects of medicine, but the basic principles of physiology are also well demonstrated and taught by it [2]. The objectives of such laboratory exercises are strictly defined by the Medical Council of India and the respective state medical universities have endorsed the same [3].

In our institution, during hematology practicals, a student has to collect his/her own capillary blood sample by pricking the finger with a hypodermic needle. Most of the government-sponsored medical teaching institutions in the country do not provide safety lancets and hence the students are compelled to use hypodermic needles. These needles are widely unpopular among students due to the potential hazards (pain, fear, anxiety) associated with them [4,5]. For a newly joined undergraduate student, fear of needles due to needle prick or belonephobia can be very stressful. It has a very high negative impact on the psychosocial well-being of students. Moreover, due to fear and anxiety, there is improper pricking of the fingers, and this can lead to faulty results as the tissue fluid can dilute the blood sample. According to existing medical literature, the penetration depth is comparatively less while pricking with a lancet in comparison to that of a hypodermic needle, and hence the degree of tissue injury and lancing pain is also minimal [6].

Very few studies have addressed these concerns of finger pricking among medical students [7], yet none from the Indian scenario exists. This study aims to compare any difference in fear and anxiety associated with finger pricking with a hypodermic needle and a safety lancet.

## Materials and methods

This is a prospective cohort study. The current study included data from first-year undergraduate medical students at the College of Medicine and Sagore Dutta Hospital for the academic years 2021-22. This study was conducted in June 2022, at the hematology practical laboratory of the Department of Physiology. Consenting students between the age groups of 17 and 25 years old with a normal body mass index were included. Participants who received regular injections for any reason, were hypersensitive to needle pricks, had a callus, scar, or burn injury on the desired finger, had neuropathy, or had known psychiatric illness were excluded from the study. The study was approved by the Institutional Ethics Committee (Memo no. ​​CMSDH/IEC/299/04-2022, dated 02/04/2022). The work has been reported in line with the Strengthening the Reporting of Observational Studies in Epidemiology (STROBE) criteria [8].

Sampling

Stratified random sampling was performed among the eligible participants (n=121) to equally divide male and female participants in both the intervention and control cohorts. The subjects could not be blinded because the practical session took place at the same venue and time. However, the data analyst was blinded by the intervention received by each participant.

Study tool

A digitized semi-structured questionnaire based on various factors associated with fear of needles and anxiety was sent to the participants of the study in the format of a Google form via electronic mail. These questions were adapted and framed to assess the subject’s behavior towards needles, type of fear, and associated factors like the smell in the room, seeing other students undergoing pricking, etc. The pain was assessed based on a numerical pain scale ranging from zero (no pain) to 10 (worst pain) [9]. We have validated the content of the questionnaire in a pilot study with 12 volunteers. Two independent faculties verified the relevance of the questionnaire. Cronbach's alpha for factors associated with needle phobia was found to be 0.71.

Study technique

After explaining the purpose of the study, informed written consent was obtained from all the participants. A demonstration session was conducted to familiarize the students with the use of routine 23/24 gauge hypodermic needles (control cohort), or safety lancets (28 gauge) (intervention cohort) for performing the experiments. After cleaning the fingers with an alcohol swab and letting them dry, the subjects were instructed to prick the pulp space (3 to 5 mm lateral to the nail bed) of the ring finger of the non-dominant hand to maintain uniformity in the study. The participants were instructed to wipe off the first drop of blood, and the subsequent free-flowing blood was collected for the experiments. The questionnaire was sent after conducting the practical. Anonymous responses from both groups were recorded on this occasion and analyzed to determine the actual prevalence of needle phobia in these students, as well as any differences in using safety lancets instead of hypodermic needles.

Statistical analysis

Quantitative variables are expressed as mean ± standard deviation or median with range. Dichotomous variables are expressed as a percentage. Intergroup differences in qualitative data were identified by chi-squared or Fisher exact tests. The strength of association between two variables was measured by logistic regression. The odds ratio at a 95% confidence interval was also calculated. A two-sided P-value of 0.05 was considered significant for each test. All statistical computations will be performed using IBM SPSS, version 20 (IBM Corp, Chicago, Illinois, USA).

## Results

A total of 121 students participated in the study. Eighty-two (67.8%) of the participants were male, and 39 (32.2%) were female. 41/82 male participants and 20/39 female participants were allocated to the intervention cohort. 111 (91.7%) pricked their finger by themselves and 10 (8.3%) asked others to prick. This was significantly higher (p=0.001) in the control cohort (n=10) compared to the intervention cohort (n=0).

Factors associated with fear and anxiety associated with needle pricks are described in Tables 1-2. The sight of others pricking during the experiment (17 versus 5, p=0.004) and the thought of pain while pricking (36 versus 16, p<0.001) was significantly associated more with the use of a hypodermic needle compared to a safety lancet. There was no significant difference in students perceiving the smell of the hematology laboratory (4 versus 1, p=0.165) and the sight of blood (9 versus 3, p=0.064) as a factor influencing their fear and anxiety in both cohorts. There was no gender difference in the perception of these factors.

**Table 1:** Frequency of variables associated with fear of finger prick and symptoms associated with finger prick. *p<0.05.

Variables associated with fear of finger prick	Needle exposure (n=60)		Lancet exposure (n=61)		P-value
	n	%	n	%	
Self-pricking during the experiment	50	83.3	61	100	0.001*
Thought of pain while pricking causes fear	36	60	16	26.2	0.001*
The smell of the hematology laboratory is a fear factor	4	6.7	1	1.6	0.165
Hearing supervisors talking about finger-prick causes fear	12	20	4	6.6	0.029*
Watching other students prick causes fear	17	28.3	5	8.2	0.004*
Site of blood creates fear	9	15	3	4.9	0.064
Symptoms associated with a finger prick					
The overall experience of any symptoms	20	33.3	8	13.1	0.008*
Dry mouth	11	18.3	1	1.6	0.002*
Shortness of breath	1	1.7	0	0	0.311
Palpitation	6	10	0	0	0.011*
Excessive sweating	16	26.7	6	9.8	0.016*
Light-headedness/feeling faint	3	5	0	0	0.077

**Table 2:** Factors associated with symptoms of fear of finger prick. *p<0.05.

Factors associated with symptoms of fear of finger prick	Univariate analysis				Multivariate analysis		
	Symptomatic		Odds ratio	P-value	B	Odds ratio	P-value
	n	%					
Female sex (n=39)	10	25.6	1.23	0.653	0.431	1.539	0.476
Intervention (subjects using safety-lancets, n=61)	8	13.1	0.30	0.008*	−0.476	0.621	0.461
Self-pricking during the experiment (n=111)	21	18.9	0.10	0.001*	1.451	4.268	0.218
Thought of pricking causes fear (n=52)	23	44.2	10.15	0.001*	1.340	3.818	0.038*
The smell of the hematology laboratory is a fear factor (n=5)	4	80	15.33	0.01*	1.678	5.357	0.256
Hearing supervisors talking about finger-prick causes fear (n=16)	7	43.8	3.11	0.053	0.542	1.720	0.467
Watching other students prick causes fear (n=22)	17	77.3	27.20	0.001*	3.055	21.214	0.002*
Site of blood creates fear (n=12)	8	66.7	8.90	0.001*	0.109	1.115	0.917

Symptoms associated with fear and anxiety related to the procedure were similar in both males and females (18 versus 10, p=0.65). These were significantly lower in the intervention cohort compared to the control cohort (8 versus 20, odds ratio 0.302, p=0.008). The most common symptom experienced was excessive sweating (n=22, 18.18%), followed by drying of the mouth (n=12, 9.91%).

**Figure 1:**
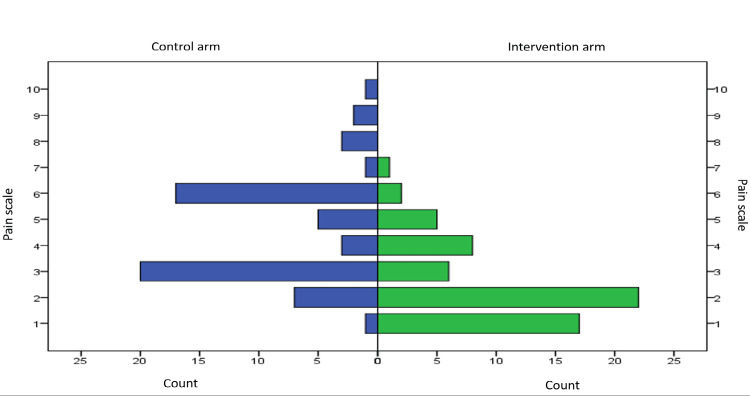
Graphical representation of the distribution of pain score between the intervention and the control arm (p=0.001).

The grading of pain is classified in Table 3. The distribution of pain scores is shown in Figure 1. There was a significant difference in pain scores between the intervention and the control cohorts. There was no significant difference in pain scores among male and female subjects.

**Table 3:** Pain score during the experiment (p=0.004).

Grading of pain	Needle exposure (n=60)		Lancet exposure (n=61)	
	n	%	n	%
Mild (0–3)	28	46.7	45	73.8
Moderate (4–6)	25	41.7	15	24.6
Severe (7–10)	7	11.7	1	1.6

## Discussion

Our study aimed to establish the difference in pain and associated fear and anxiety experienced by undergraduate medical students while performing hematology practicals using hypodermic needles or safety lancets. In contrast to other contemporary studies [4,10-12], there was no gender difference in fear responses and anxiety.

Fear acquisition with a traumatic experience in the healthcare setting is still a debate [13]. An insightful study by Van Wijk et al. revealed a relationship between early experiences of injection and anxiety with mandibular block [14]. Andrews et al. established the relationship between hospitalization and fear responses to clinical procedures with needles [15]. Unfortunately, this was not screened in the present study. We excluded the participants who received regular injections for any reason from the current study to eliminate any potential subject bias.

In our study, fear of pain and associated symptoms were significantly lower in the intervention arm who had done the hematology experiment with a safety lancet compared to the control group. There was also a higher frequency of mild grades of numeric pain scores and a lower frequency of moderate and severe grades of pain scores in the intervention arm. This could be due to a lesser depth of penetration with thinner and shorter safety lancets compared to wider (22/23 G) and longer hypodermic needles. A lesser depth of penetration causes lesser tissue injury and hence lesser pain [16,17].

In a study by Roy et al., it was shown that subjects who tend to prick the sides of their fingers tend to feel a greater degree of lancing pain compared to those who pricked the middle part of the finger pulp [18]. This was highly subjective as no proper demarcation was made to distinguish the side and middle part of the finger. To eliminate this confounding factor, we standardized the experiment with prior demonstrations conducted by the faculty before the actual experiments.

The smell of hematology practicals mainly comes from the alcohol, phenol, and other reagents used. This could be a stressor for the experiment. Unlike previous studies [10], in our study, there was no difference in perception of this as a fear factor among the intervention and control cohorts. This could be due to repeated exposure of the students to the hematology practical laboratory prior to the index experiment (there is a weekly one hematology practical session).

Many students (n=22, 18.1%) have reported that the sight of their peers performing the experiment is a risk factor associated with fear and anxiety. This could be attributed to the facial grimace observed due to pain. Similar studies have shown a significant association between fear factors and others receiving vaccines [10,19]. In a study by Willershausen et al., 47% of the respondents reported panic at the sight of an injection needle prior to receiving medication [20]. A significant number of subjects reported the sight of blood as a fear factor, but there was no significant difference between the intervention and control cohorts.

Stress symptoms (dry mouth, palpitation, shortness of breath, excessive sweating, and fainting) secondary to fear factors, anxiety, and pain were reported in 28 (23.1%) participants. This was significantly higher in the control cohort compared to the intervention cohort (8 versus 20, odds ratio 0.302, p=0.008). This constellation of symptoms may be due to autonomic response, which can ultimately lead to vasovagal syncope [21].

The cost of a hypodermic needle in India ranges from Rupees 0.90 to 1.75 (0.012-0.022 USD), whereas that of a safety lancet ranges from Rupees 0.965 to 1 (0.012-0.013 USD). Both of these are comparable and can be used interchangeably without much alteration in budget allocation.

The study has some strengths and limitations. The study's strength is that it is the only single-institution prospective cohort study addressing this important issue. To the best of our knowledge, this is the only study reported from India where the quality of medical education, as well as emotional well-being as a factor affecting the quality of medical education in India, is discussed and reviewed. The drawbacks are: (i) It is a single-center study. (ii) Blinding of subjects could not be performed in the scenario that could yield subject bias. (iii) We did not screen the subjects for an earlier traumatic experience, which can give rise to bias. (iv) A cost analysis study was not done in greater detail. (v) The gender distribution was uneven.

Future studies, preferably multicenter studies with a focus on cost-effective analysis, should be done to assess the superiority of one modality over the other.

## Conclusions

This study compares and contrasts the differences in fear and anxiety associated with finger pricking with hypodermic needles and safety lancets. Since a considerable difference between the two was found, the use of lancets can be proposed to the medical teaching institutions for psychological advantage, pain reduction, and overall better quality of the process. Moreover, the emotional component of a medical student has never been considered an important factor in medical education. This would be a remarkable quality improvement project in terms of health education.

## References

[1] Latif H, Majoka MI, Khan MI (2017). Emotional intelligence and job performance of high school female teachers. Pakistan J Psychol Res.

[2] Savitha D, Taniya A (2020). Student perceptions of "doing" hematology physiology practicals. Adv Physiol Educ.

[3] Medical Council of India (2018). Regulations on graduate medical education. https://www.nmc.org.in/rules-regulations/graduate-medical-education-regulations-1997/.

[4] Deacon B, Abramowitz J (2006). Fear of needles and vasovagal reactions among phlebotomy patients. J Anxiety Disord.

[5] Hanas R (2004). Reducing injection pain in children and adolescents with diabetes: a review of indwelling catheters. Pediatr Diabetes.

[6] Veglio M, Sivieri R, Trento M, Porta M (1996). Finger-pricking devices: are they less painful than lancets?. Diabet Med.

[7] Milovanović B, Tomović D, Janković SM (2017). Factors influencing the fear of needles amongstudents of medicine and pharmacy. Acta Fac Med Naissensis.

[8] von Elm E, Altman DG, Egger M, Pocock SJ, Gøtzsche PC, Vandenbroucke JP (2007). The Strengthening the Reporting of Observational Studies in Epidemiology (STROBE) statement: guidelines for reporting observational studies. Ann Intern Med.

[9] Snyder D (1992). Pain: clinical manual for nursing practice. By Margo McCaffery and Alexandra Beebe. St. Louis: Mosby Company, 1989, 353 pp., 25.95 (softcover). Cancer Nurs.

[10] Nir Y, Paz A, Sabo E, Potasman I (2003). Fear of injections in young adults: prevalence and associations. Am J Trop Med Hyg.

[11] Jacobson RM, Swan A, Adegbenro A, Ludington SL, Wollan PC, Poland GA, Vaccine Research Group (2001). Making vaccines more acceptable—methods to prevent and minimize pain and other common adverse events associated with vaccines. Vaccine.

[12] Banerjee I, Roy B, Sathian B, Banerjee I, Kumar SS, Saha A (2010). Medications for anxiety: a drug utilization study in psychiatry inpatients from a tertiary care centre of Western Nepal. Nep J Epid.

[13] Poulton R, Menzies RG (2002). Non-associative fear acquisition: a review of the evidence from retrospective and longitudinal research. Behav Res Ther.

[14] van Wijk A, Lindeboom JA, de Jongh A, Tuk JG, Hoogstraten J (2012). Pain related to mandibular block injections and its relationship with anxiety and previous experiences with dental anesthetics. Oral Surg Oral Med Oral Pathol Oral Radiol.

[15] Andrews GJ (2011). 'I had to go to the hospital and it was freaking me out': needle phobic encounter space. Health Place.

[16] Fruhstorfer H, Müller T, Scheer E (1995). Capillary blood sampling: how much pain is necessary? Part 2: relation between penetration depth and puncture pain. Pract Diab Int.

[17] Garvey K, Batki AD, Thomason HL, Holder R, Thorpe GH (1999). Blood lancing systems for skin puncture. Prof Nurse.

[18] Roy B, Sathian B, Banerjee I, Khan I (2014). Belonephobia and finger pricking associated pain in hematology laboratory: A cross sectional study among undergraduate medical students in Nepal. Nep J Epid.

[19] Ost LG, Sterner U, Lindahl IL (1984). Physiologic responses in blood phobics. Behav Res Ther.

[20] Willershausen B, Azrak A, Wilms S (1999). Fear of dental treatment and its possible effects on oral health. Eur J Med Res.

[21] Ritz T, Meuret AE, Ayala ES (2010). The psychophysiology of blood-injection-injury phobia: looking beyond the diphasic response paradigm. Int J Psychophysiol.

